# On the Beneficial Effects upon Intractaele Strictures of Opening the Urethra behind Them

**Published:** 1857-06

**Authors:** 


*On the Beneficial Effects upon Intractable Strictures of opening the Urethra behind them.*—The benefits of the plan of treating bad strictures by procuring a free escape for the urine behind the strictured tract has been well illustrated by a case now under the care of Mr. Cock, in Guy's Hospital. The end referred to may be secured either by puncture of the bladder, or by opening the membranous urethra, preference for one or the other mode being often optional with the surgeon. Of course, when no retention has occurred, and the bladder is kept empty, puncture of it can be resorted to; and in this class of cases, as well as those in which the perineum is the seat either of abscesses or sinuses, the perineal operation is to be preferred. The clinical fact on which the practice is founded is, the strictures, when once relieved of the irritation of the urine passing over them, spontaneously soften down and dilate. The plan, although much ridiculed by some, is a great favorite with some of our most practical hospital surgeons, among whom we may mention Mr. Cock, Mr. Wormald, and Mr. Simon. Mr. Wormald terms it giving "the diseased urethra a holiday," and is very warm in his commendations. Mr. Cock and Mr. Simon we have often seen resort to it. In cases of impassable stricture, its advocates regard it as far safer practice than the attempt to cut through the stricture without a guide, a procedure acknowledged to be one of much difficulty and uncertainty. Mr. Cock's case referred to, may be briefly outlined as follows: A man in fair health, aged 30, had been for five years the subject of a long and most intractable stricture. His penis, &c., at the time of admission, was seamed with scars of incisions made for the relief of extravasation, and he stated that he had repeatedly suffered attacks of retention. The urine merely dribbled away, and there were numerous fistulæ in the perineum. After some weeks' patient treatment, Mr. Cock still found himself unable to pass the smallest instrument. The mass of induration around the urethra was very large. The man was now put under chloroform, and with a straight-bladed knife Mr. Cock opened the urethra by a single plunge,* just anterior to the prostrate. No staff was in the urethra at the time, and no attempt

* The term "plunge" may possibly be understood. All that it is intended to imply is, that the knife should be pushed steadily on in the median line; until the urethra is reached, and that there should be no side cutting. The forefinger of the left hand should be in the rectum, just touching the anterior edge of the prostate, and should serve in a certain sense as a guide to the knife. The making but one cut with the knife secures that the wound shall be of a conical shape, its apex being the urethra. The circumstance that the urethra is dilated behind the stricture much facilitates the finding of it. The careful keeping the knife in the median line is the one important point in the operation.

whatever was made to divide the stricture. A female catheter was passed by the wound into the bladder and retained a few days. No urine whatever flowed by the penis for upwards of a fortnight, and at the end of this time a No. 5 catheter was pretty readily introuced through the stricture. No case could better prove the principle that strictures, when relieved from irritation, do really soften and spontaneously dilate. There is, indeed, whatever some may have held, nothing singul r in the circumsiance that they should do so. In cases of Amussat's operation, performed for stricture of the rectum, the latter has been known to dilate afterwards very considerably, and the effect of tracheotomy in relieving chronic laryngeal disease, is well known to all surgeons.—*Med. Times and Gazette*, Dec. 24, 1856.

